# A rare case of left‐sided obturator hernia diagnosed by computed tomography

**DOI:** 10.1002/ccr3.7297

**Published:** 2023-05-09

**Authors:** Manisha Aryal, Suraj Keshari, Archana Pandey, Abhishek Pandey, Ishwor Paudel

**Affiliations:** ^1^ Kathmandu University School of Medical Sciences Dhulikhel Hospital Dhulikhel Nepal; ^2^ National Academy for Medical Sciences Bir Hospital Kathmandu Nepal

**Keywords:** case report, computed tomography, obstructed hernia, obturator hernia, Richter's hernia

## Abstract

Obturator hernia is a rare condition that is difficult to detect clinically. We highlight the importance of a CT scan in establishing an early diagnosis of an obturator hernia, effective surgical intervention planning, thereby enhancing the result.

## Graphical abstract

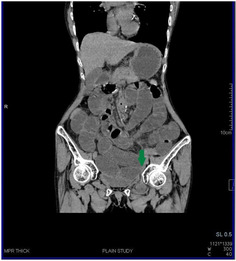

## 1 INTRODUCTION

Obturator hernia is a rare type of abdominal hernia that is a significant cause of morbidity and mortality, particularly in the elderly.^1^ An obturator hernia arises when intraperitoneal or extraperitoneal contents protrude through the obturator canal.^2^ Obturator hernia was first identified in 1724, and it is estimated to account for 0.05%–0.14% of all hernias and 0.2%–1.6% of all small bowel obstructions.^3^ This uncommon hernia is frequently observed in thin, elderly, multiparous women.^4^ Because it is rarely externally apparent or palpable, it frequently goes unnoticed or misdiagnosed.^5^ Computed tomography (CT) is increasingly being used to detect obturator hernia in cases of intestinal obstruction, greatly assisting surgeons in emergency management.^6^

## 2 CASE DETAILS

A lady in her 70s was brought to the emergency department after experiencing significant abdominal pain in the left lower quadrant for 3 days. It was associated with constipation and numerous bouts of vomiting. She has been a smoker since she was 15 years old, averaging 5–10 cigarettes per day. She has a medical history of chronic obstructive pulmonary disease and has been taking medication for 7 months with poor compliance. She had a history of hemorrhoidectomy surgery around 2 years ago. She has given birth eight times, with two abortions. On examination, her heart rate was 122 beats per minute, and her blood pressure was 110/80 mmHg. The abdomen was soft and tender in the lower left quadrant, with no guarding or rigidity. Femoral and inguinal hernias were not detectable on palpation. On chest auscultation, both sides had decreased air entry.

## 3 INVESTIGATIONS

**FIGURE 1:**
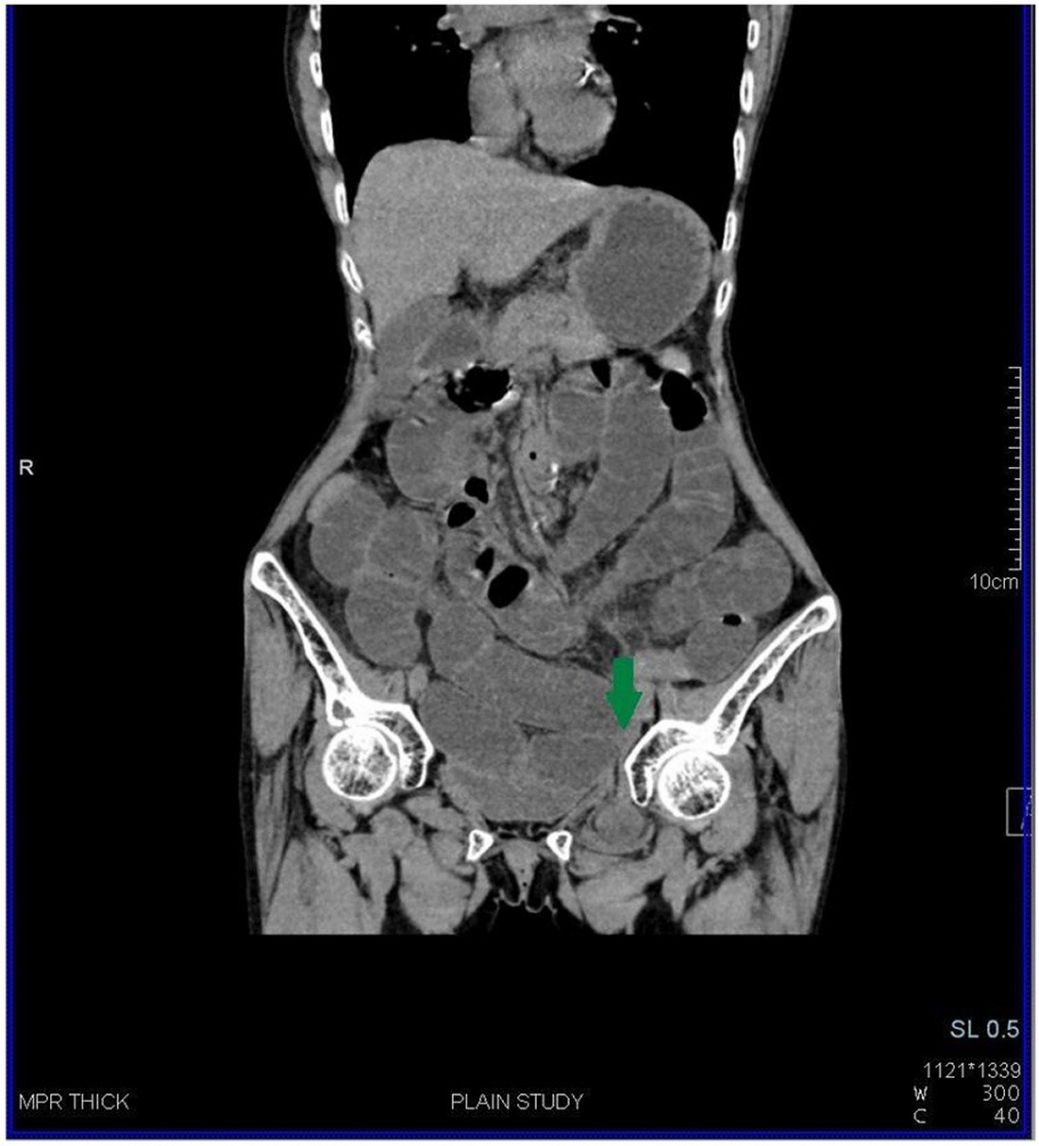
This is a plane coronal computed tomography showing left obturator hernia and fluid filled dilated proximal small bowel loops with maximum luminal diameter of 3.6 cm with collapsed terminal ileum and large bowel loops.

**FIGURE 2:**
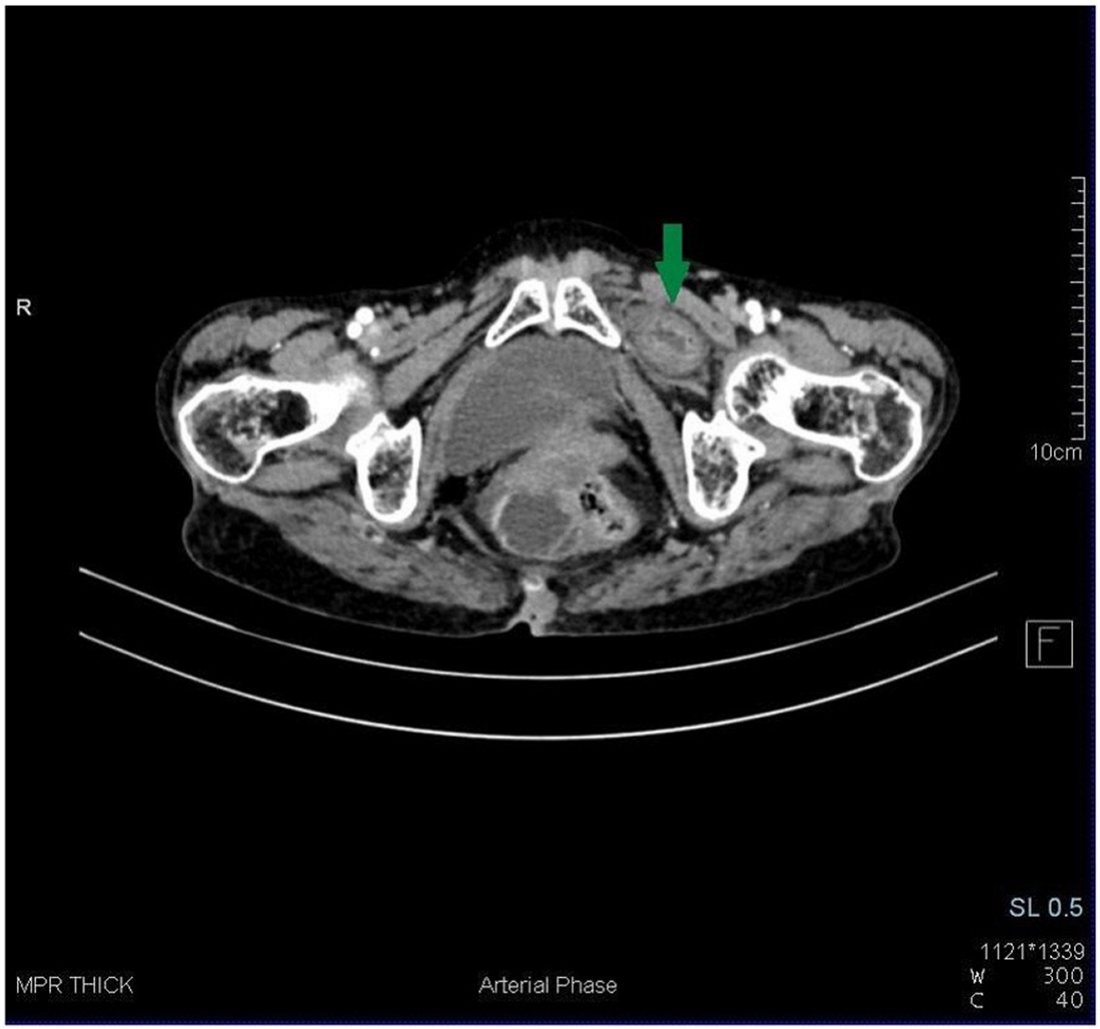
Arterial phase axial computed tomography showing a loop of small bowel (distal jejunum) herniating through the left obturator foramen between the left pectineus and obturator externus muscles. On the opposite side, three muscular layers are clearly visible around the obturator foramen: internal obturator, external obturator, and pectineus.

Blood tests were performed and found to be within normal limits. Ultrasonography revealed features of small bowel obstruction and ascites. Her CT scan demonstrated a loop of small bowel (distal jejunum) herniating through the left obturator foramen between the left pectineus and obturator externus muscles (Figures 1 and 2). Fluid filled dilated proximal small bowel loops with maximum luminal diameter of 3.6 cm with collapsed terminal ileum and large bowel loops were noted (Figure 1). Emphysematous changes in bilateral basal lung fields were also noted.

## 4 TREATMENT

**FIGURE 3:**
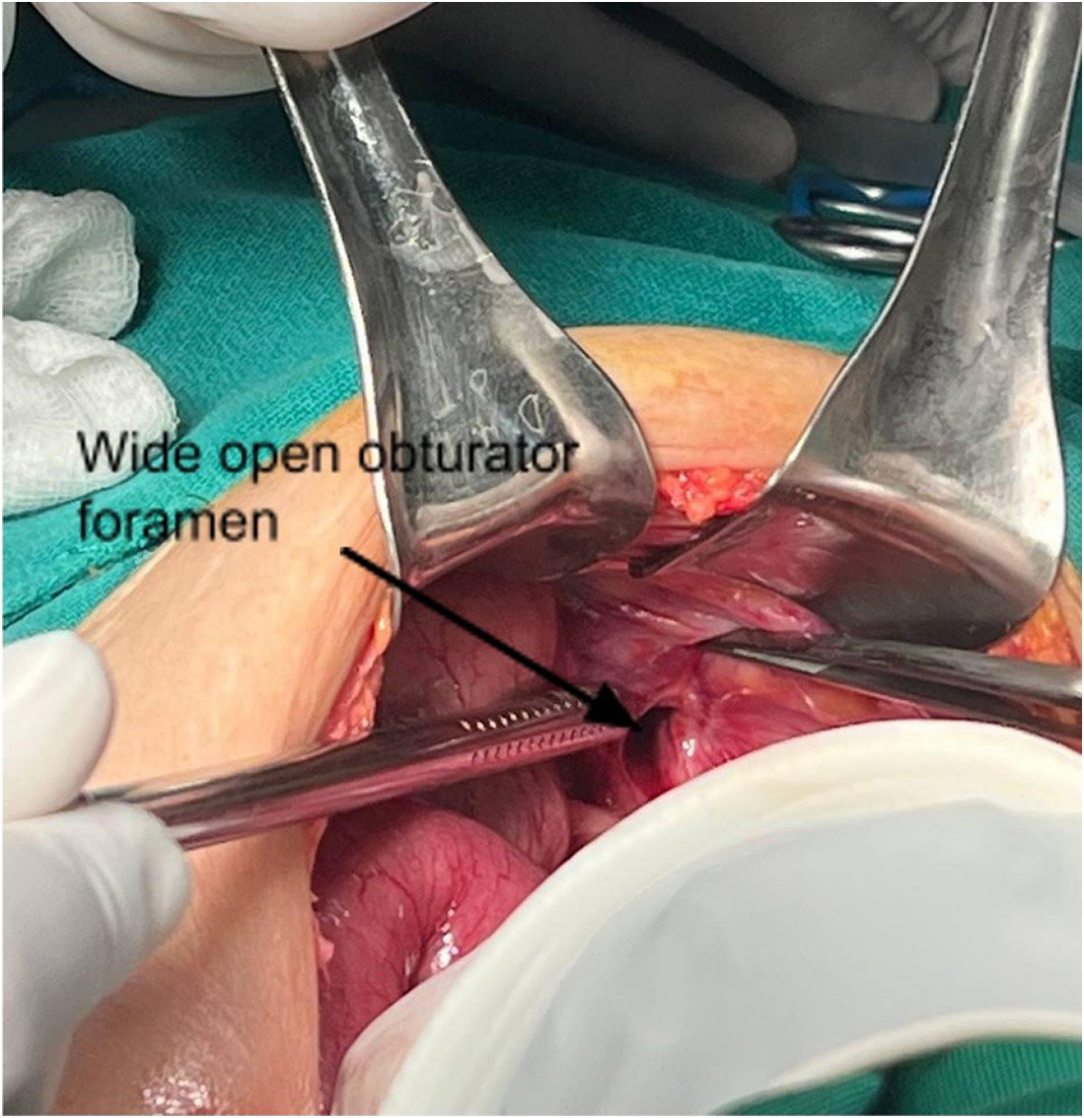
Intraoperative image showing wide open left obturator foramen.

**FIGURE 4:**
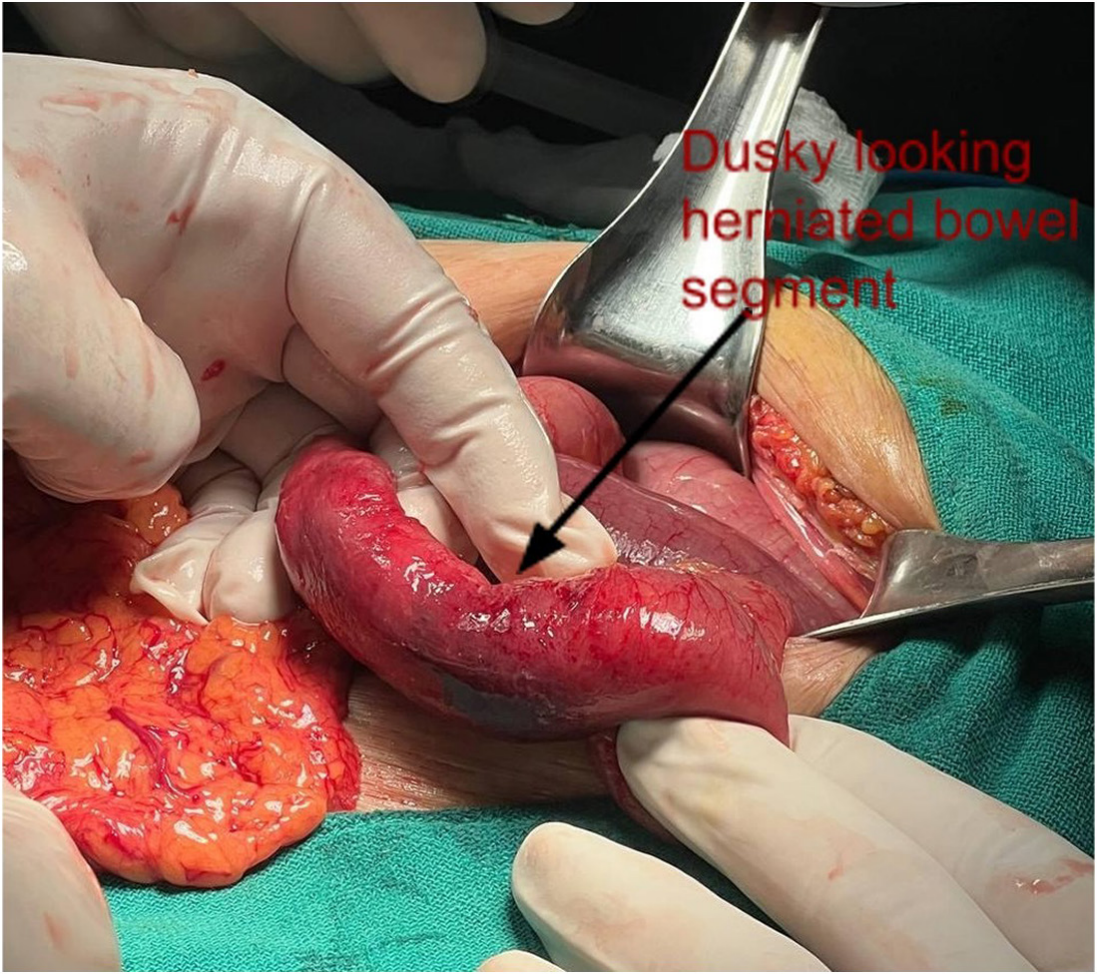
Dusky appearance of herniated bowel loop signifying ischemic changes.

She had an emergency laparotomy, which revealed wide open left obturator foramen (Figure 3) with herniation of partial circumference of small bowel, Richter's type with dilated proximal bowel loop and collapsed distal bowel loop. The herniated bowel loop had dusky appearance signifying ischemic changes (Figure 4). Reduction and repair of the left obturator hernia were performed.

## 5 OUTCOME AND FOLLOW UP

Following repair of her obturator hernia, the patient's presenting symptoms resolved.

Postoperatively, she was hospitalized in the intensive care unit (ICU) for 4 days due to oxygen requirement. On day 1, 300 cc of a bluish‐green fluid was drained from her nasogastric tube. Her nasogastric tube was taken out on day 3. On day 4, she moved to the surgical ward, where she spent the next 5 days. Multidisciplinary efforts of the surgical, medical, and physiotherapy teams produced quick results.

## 6 DISCUSSION

The protrusion of abdominal viscera through the obturator canal is known as an obturator hernia. The obturator nerve and vessels are located inside this 2–3 cm long and 1 cm broad structure, which is bordered inferiorly and laterally by the obturator membrane and superiorly and laterally by the pubic bone.^1^ Corpus adiposum serves as a cushion for the obturator nerve.^1^ It is particularly prevalent in older women aged 70–90 due to loss of the protective fat (Corpus adiposum) in the obturator canal, which predisposes hernia development, hence the title “little old woman's hernia.”^5^ Women have this condition 6–9 times more frequently due to their wider pelvis and greater obturator canal than males.^3^ Pregnancy, kyphoscoliosis, chronic lung illness, constipation, chronic urinary obstruction can all increase intra‐abdominal pressure, which increases the risk of herniation.^7^ In this case, the patient had multiple pregnancies, chronic obstructive pulmonary disease, and constipation, all of which increase the risk of herniation. Because the sigmoid colon tends to prevent herniation on the left, it occurs more frequently on the right side.^3^ However, in this instance, the herniation was on the left side.

Because the signs and symptoms are frequently non‐specific, it is estimated that only 20%–30% of obturator hernia patients receive a correct preoperative diagnosis.^8^ A palpable protrusion in the obturator region (medial thigh) is typically absent due to its deep location.^9^ Pelvic CT, on the other hand, have been useful in the diagnosis of obturator hernia.^10^ According to the literature, up to 80% of patients with obturator hernias have symptoms of bowel obstruction, which is often partial due to a high proportion of patients having Richter's herniation of the bowel into the obturator canal.^10^ The Howship Romberg sign manifests as pain along the medial aspect of the thigh or knee caused by hernia sac compression of the obturator nerve within the canal, while considered pathognomonic, its sensitivity is low, and specificity varies widely.^11^ Typically, this pain is exacerbated by extension and abduction or inward rotation of thigh.

Obturator hernia has three stages. The first stage includes the entry of preperitoneal tissue into the pelvic opening of the obturator canal, whereas the second stage involves the formation of a dimple in the peritoneum and leads to the formation of a peritoneal sac overlaying the canal. During the third stage symptoms are caused by the herniation of the viscera into this sac.^1^, ^3^, ^12^

Actually, emergency multidetector CT scanning could result in more rapid diagnosis and earlier surgical intervention, improving the outcome. Meziane et al. reported the first use of a CT scan to detect obturator hernia in 1983.^13^ A herniated loop of distal small bowel extending through the obturator foramen between the pectineus and obturator externus muscles is a common CT scan finding. CT scan of an incarcerated hernia reveals associated bowel loop dilatation in the abdomen and in severe cases, the bowel becomes edematous and ischemic, leading to gangrene and perforation.^13^ According to some reports, the use of CT scan has increased the rate of preoperative diagnosis from 43% to 90%.^5^ Other diagnostic methods, such as abdominal x‐ray, herniography, ultrasonography, and gastrointestinal imaging with contrast medium, have been reported to be useful. Plain abdominal x‐ray films can show gas in the obturator region, indicating a hernia.^4^

Obturator hernia can only be treated surgically. In an emergency, an abdominal approach through a low midline incision is preferred. Because of gangrene or perforation, resection of the involved portion of bowel is sometimes required.^13^

## 7 CONCLUSION

An obturator hernia is very rare and difficult to diagnose with a high rate of morbidity and mortality. This type of hernia must be considered in elderly and chronically ill women with signs of bowel obstruction. Abdominal CT scan is the best diagnosis tool. Early CT should be taken into consideration in situations of intestinal obstruction in order to speed up the diagnosis and, as a result, reduce consequences such as bowel ischemia and the requirement for bowel resection during surgery.

## CONSENT

This patient was properly informed for her clinical information to be included in this publication. Written consent was obtained from her grand‐daughter as the patient herself could not read or write.

## AUTHOR CONTRIBUTIONS

**Manisha Aryal:** Investigation; supervision; writing – review and editing. **Suraj Keshari:** Investigation; writing – original draft; writing – review and editing. **Archana Pandey:** Writing – original draft; writing – review and editing. **Abhishek Pandey:** Writing – review and editing. **Ishwor Paudel:** Writing – review and editing.

## CONFLICT OF INTEREST STATEMENT

All authors declare that there is no conflict of interest regarding the publication of the article.

## FUNDING INFORMATION

None.

## Data Availability

Data sharing not applicable to this article as no datasets were generated or analysed during the current study.
